# Comments on potential health effects of MRI-induced DNA lesions: quality is more important to consider than quantity

**DOI:** 10.1093/ehjci/jew163

**Published:** 2016-08-22

**Authors:** M.A. Hill, P. O'Neill, W.G. McKenna

**Affiliations:** CRUK/MRC Oxford Institute for Radiation Oncology, University of Oxford, Gray Laboratories, ORCRB Roosevelt Drive, Oxford OX3 7DQ, UK

**Keywords:** MRI, DNA damage, ionizing radiation, cancer risk

## Abstract

Magnetic resonance imaging (MRI) is increasingly being used in cardiology to detect heart disease and guide therapy. It is mooted to be a safer alternative to imaging techniques, such as computed tomography (CT) or coronary angiographic imaging. However, there has recently been an increased interest in the potential long-term health risks of MRI, especially in the light of the controversy resulting from a small number of research studies reporting an increase in DNA damage following exposure, with calls to limit its use and avoid unnecessary examination, according to the precautionary principle. Overall the published data are somewhat limited and inconsistent; the ability of MRI to produce DNA lesions has yet to be robustly demonstrated and future experiments should be carefully designed to optimize sensitivity and benchmarked to validate and assess reproducibility. The majority of the current studies have focussed on the initial induction of DNA damage, and this has led to comparisons between the reported induction of γH2AX and implied double-strand break (DSB) yields produced following MRI with induction by imaging techniques using ionizing radiation. However, γH2AX is not only a marker of classical double-ended DSB, but also a marker of stalled replication forks and in certain circumstances stalled DNA transcription. Additionally, ionizing radiation is efficient at producing complex DNA damage, unique to ionizing radiation, with an associated reduction in repairability. Even if the fields associated with MRI are capable of producing DNA damage, the lesions produced will in general be simple, similar to those produced by endogenous processes. It is therefore inappropriate to try and infer cancer risk by simply comparing the yields of γH2AX foci or DNA lesions potentially produced by MRI to those produced by a given exposure of ionizing radiation, which will generally be more biologically effective and have a greater probability of leading to long-term health effects. As a result, it is important to concentrate on more relevant downstream end points (e.g. chromosome aberration production), along with potential mechanisms by which MRI may lead to DNA lesions. This could potentially involve a perturbation in homeostasis of oxidative stress, modifying the background rate of endogenous DNA damage induction. In summary, what the field needs at the moment is more research and less fear mongering.

## Introduction

Magnetic resonance imaging (MRI) is a widely used diagnostic technique capable of acquiring both anatomical and functional information on organs within the body. It is increasingly being used in cardiology to detect heart disease and guide therapy. It is mooted to be a safer alternative to x-ray and radioisotope imaging techniques, such as computed tomography (CT) or coronary angiographic imaging, where there is a clear relationship between exposure to ionizing radiation and increased cancer risk, as recently demonstrated by epidemiological studies for paediatric CT exposures.^1,2^ However, with the increase in the clinical use of MRI, there has also been an increased interest in the potential risks, especially in the light of the controversy resulting from a limited number of studies reporting an increase in DNA damage induction following exposure,^3–5^ with calls to limit its use and avoid unnecessary examination, according to the precautionary principle.^6–9^

MRI results in exposure of the patient to a mixture of static magnetic fields (SMF), time-varying gradient magnetic fields (GMF) typically in the range 10–100 kHz, and pulsed radiofrequency fields (RF) in the MHz range. While for occupational exposures of staff working with or in close proximity to the scanner, SMF is the most relevant. The known risks of using MRI mainly relate to the interaction of magnetic and radiofrequency fields with ferromagnetic and paramagnetic objects (limiting its use on patients with metal objects within the body) and that movement of a person through a strong SMF may induce effects such as vertigo and nausea.^10^ In addition, RF may cause some heating of tissue, and GMF are capable of stimulating nerves and muscles; however, in practice, the operation limits are set to minimize these effects. With regard to biological and long-term health effects associated with MRI exposure and its various components, a number of comprehensive reviews have been published by national and international committees^10–16^ along with a recent review of genotoxicity associated with MRI exposures.^4^ Although the data are somewhat limited, the current consensus is that no clear link exists between MRI or associated magnetic and pulsed radiofrequency fields and subsequent health risks. The relevant data are discussed below. We conclude that it is essential to avoid over interpretation of the limited data available on potential genetic effects of MRI and making uninformed comparisons of these effects with the kinds of DNA damage produced by ionizing radiation.

## Does MRI exposure lead to DNA damage?

The potential of adverse effects associated with exposure to magnetic fields has been studied for a number of years both *in vitro* and *in vivo*. DNA damage, especially DNA double-strand break (DSB) and chromosome aberrations (CA), has typically been a main focus of research, since DSB and CA have the greatest potential to lead to carcinogenesis or hereditary effects. The effects of exposure to individual fields, SMF, GMF, or RF have been extensively reviewed by various national and international committees^10–16^ who typically concluded that there is no clear link between exposure and an increase in genetic damage. However, current MRI exposures are a complex combination of SMF, GMF, and RF. Only a very limited number of investigations of genetic damage using clinical MRI scanners, and the types of sequences routinely used in the clinic, have been published and are briefly summarized in *Table 1*. Seven of these studies were also recently critically reviewed by Vijayalaxmi *et al.*^4^ What is clear is that the experimental data relating to MRI genotoxicity are limited and inconsistent.

**Table 1:** Investigations of genetic damage using clinically relevant scanners or pulse sequences

	Schreiber *et al.*^17^	Schwenzer *et al.*^18^	Simi *et al.*^9^	Lee *et al.*^19^	Yildiz *et al.*^20^	Fiechter *et al.*^8^	Szerencsi *et al.*^21^	Lancellotti *et al.*^7^	Brand *et al.*^22^	Reddig *et al.*^23^
Assay	Mutation (Ames test)	DSB (γH2AX: FC and foci)	MN	CA, MN, and SSB (alkaline comet)	SSB (alkaline comet)	DSB (γH2AX: FC and foci)	SSB (alkaline comet); MN	DSB (γH2AX: FC)	DSB (γH2AX: foci)	DSB (γH2AX: FC and foci)
Study	*In vitro*	*In vitro*	*In vitro* and *in vivo*	*In vitro*	*In vivo*	*In vivo*	*In vitro*	*In vivo*	*In vivo*	*In vitro*
Flux	1.5 T and 7.2 T	3 T	1.5 T	3 T	1.5 T	1.5 T	3 T	1.5 T	1.5 T	7 T
Scan protocol	Static only; static (1.5 T) + time-varying bipolar GMF; static (1.5 T) + pulsed RF	Static only; Static + turbo spin-echo (TSE); Static + gradient-echo (GE)	Cardiac	Brain: range of pulse sequences	Hypophysial	Cardiac	Brain: range of pulse sequences	Cardiac: range of pulse sequences	Cardiac: range of pulse sequences	Static only; Static with varying GMF and pulsed RF
Scan duration	1.5 sT: 1 h and 24 h 7.2 T: 1 h	TSE: 2 min 20s or 2 h	*In vitro*: 686, 1186, 1618, 2188s	22, 45, 67, 89 min	∼16 min	68 ± 22 min	22, 45, 67, 89 min	35–40 min	30–60 min	1 h
Contrast agent	–	–	No contrast agent	–	With and without gadolinium	Gadolinium	–	No contrast agent	Gadolinium	–
Cells	*Salmonella typhimurium* bacteria	Human cancer cells (HL-60 and KG-1a)	Human blood lymphocytes	Human blood lymphocytes	Human blood lymphocytes	Human blood lymphocytes	Human blood lymphocytes	Human blood (T lymphocytes and NK cells)	Human blood lymphocytes	Human blood lymphocytes
Expts/Donors	≥2 expts	–	*In vitro*: 8 healthy donors *In vivo*: 8 patients and volunteers	Single healthy donor	28 patients	20 patients	2 healthy donors/3 repeats	20 healthy donors	45 patients	16 healthy donors
Temp	1.5 T: 32–37°C 7.2 T: 37°C	37°C	*In vitro*: Room temp *In vivo*: body temp	25°C	Body temp	Body temp	20°C	Body temp	Body temp	–
Assay time points	Colonies counted after 48 h	0, 1, and 24 h post imaging	PHA stimulation: *In vitro* at 0 h and 24 h post scan; *In vivo* at 0, 24, 72, 96, 120 h post scan	PHA stimulation prior to exposure. SSB: 0 h post exposure MN: 0 h CA: 0 h	0 h post non-contrast scan; 0 h post subsequent contrast-enhanced scan	0 h post imaging	SSB: 0 h post exposure; MN: 0 h PHA stimulation (following 20 min transport)	1 h, 2 h, 2 days, 1 month and 1 year post imaging	5 min post imaging	0, 1, 20 h post imaging
Positive control	Chemical mutagens	4 Gy 6MV x-rays	–	SSB: cisplatin MN, CA: bleomycin	–	–	4 Gy γ-ray	–	–	120 kV CT scan and 0.2 Gy γ-rays
Results	No mutagenic or co-mutagenic effect observed	No significant increase observed	*In vitro*: Significant increase at 0 h, frequency increasing with exposure time. Reduced after 24 h (2 highest exposures still significant) *In vivo*: Significant increase after 0 h and 24 h but not later times	SSB, MN, CA: significant increase with increasing exposure times of 45 min and above	No significant increase after non-contrast-enhanced MRI; Significant increase following subsequent contrast-enhanced MRI	DSB (FC, foci): Significant increase observed	SSB, MN: No significant increase observed	T lymphocytes: 1 h, 2 h, 1year—no significant effect; 2 days, 1 month—significant increase; NK cells: a slight increase was observed at 2 h and day 2	No significant increase observed	No significant increase observed

SSB, assessed using the alkaline comet assay; DSB, assessed using γH2AX measured using either flow cytometry (FC) or immunofluorescent microscopy and counting resultant foci (foci); MN assay, following stimulation with phytohaemagglutinin (PHA), cells incubated for 72 h, with cytochalasin-B added after 44 h; CA assay, following stimulation with PHA, cells incubated for 48 h, with colcemid added after 45 h.

### DNA single-strand breaks

Three of the studies investigated the induction of single-strand breaks (SSB) using the alkaline comet assay. The study of Szerencsi *et al.*^21^ showed no enhancement immediately following *in vitro* exposure of human leukocytes. In contrast, statistically significant enhancements were observed by Lee *et al.*^19^ following *in vitro* exposure times of 45 min or greater, with the observed level increasing with duration of exposure. For *in vivo* exposures, Yildiz *et al.*^20^ observed no significant increase in lymphocytes taken after the initial non-contrast-enhanced MRI. However, they did report a significant increase in the lymphocytes taken following the subsequent contrast-enhanced MRI.

### DNA double-strand breaks

Five studies investigated DSB induction using γH2AX assays (a DSB along with several other modifications results in the phosphorylation of surrounding H2AX histones). Three of the studies showed no enhancement of DSB following either *in vitro* exposure of human cancer cell lines 0, 1, or 24 h post exposure,^18^ *in vitro* exposure of human lymphocytes 0, 1, or 20 h post exposure,^23^ or in human lymphocytes taken 5 min after *in vivo* exposure of patients undergoing contrast-enhanced cardiac magnetic resonance imaging (CMR).^22^ In contrast, Fiechter *et al.*^8^ reported a statistically significant enhancement in DSB in human lymphocytes taken from patients immediately following contrast-enhanced CMR. While Lancellotti *et al.*^7^ reported no enhancement in T lymphocytes taken from volunteers 1 and 2 h post exposure to unenhanced CMR, but significant enhancement was reported at day 2 and 1 month post exposure before returning to base line levels after 1 year (a significant increase was observed at 2 h and day 2 for NK cells but not at other time points).

### Chromosome aberrations/micronuclei

While Szerencsi *et al.*^21^ showed no increase in the yield of micronuclei (MN) following *in vitro* exposure of human lymphocytes, a significant enhancement of both MN and CA (using Giemsa staining) was reported by Lee *et al.*^19^ for *in vitro* exposure times of 45 min and greater, with the observed level increasing with duration of exposure. The most frequent types of aberrations observed were chromatid breaks, with both exchange-type and deletion-type CA being observed for the longest exposure time (89 min). A significant increase in MN frequency was also reported by Simi *et al.*^9^ in lymphocytes at 0 h post *in vitro* exposure, with the frequency increasing with increasing duration of exposure. While for the two shorter exposures no significant increase in MN frequency above controls was observed for cells left for 24 h to repair before performing the assay, a significant increase was still observed for the two longer exposure times. Following *in vivo* exposure, a significant increase was observed for all participants when blood was collected immediately or 24 h post exposure, although no difference was observed at later collection times (72, 94, and 120 h).

### Mutations

Single experiment using the Ames test shows no mutagenic or co-mutagenic effect.^17^

## DNA assays and relevance of DNA lesions to cancer?

Cells have several well-developed DNA-repair pathways and DNA-damage checkpoints to deal with the frequent DNA lesions produced by endogenous or exogenous sources and the vast majority of lesions will be repaired. However, the efficiency and fidelity of repair, and therefore the relevance, will be dependent on the type of lesion produced. Accurate quantification of these lesions is also not trivial, especially at low levels; therefore, great care must be taken to ensure consistency and with appropriately interpreting the results.

The standard alkaline comet assay detects strand breaks along with alkali-labile sites, although the technique can also be modified to visualize the presence of base damage by using lesion-specific endonucleases that convert base damage into SSB.^24^ However, these lesions are typically repaired quickly with high fidelity and so are much less likely to be relevant to long-term health effects compared with DSB.^25,26^

DNA DSB and particularly complex DSB (see below) are considered to be particularly important with respect to carcinogenesis as they are intrinsically more difficult to repair than other types of DNA damage with their potential to lead to modification, rearrangement, or loss of chromosomal material.^27^ An early response to DSB is the phosphorylation of the histone H2AX, which can be detected using a fluorescent antibody in conjunction with immunofluorescent microscopy to visualize discrete nuclear foci at sites of DSB.^28,29^ Radiation-induced foci have been observed following exposure to ionizing radiation with doses as low as 1 mGy in stationary phase human fibroblasts (∼0.02–0.04 DSB per cell), with the yield increasing linearly with dose.^30^ Studies have also demonstrated that the yield of γH2AX foci can be a useful quantitative biomarker of human low-level ionizing radiation exposure in blood taken from an exposed individual at doses down to 6 mGy.^31^ This technique has significantly greater sensitivity in detecting DSB than direct methods which require many hundreds of DSB per cell, but it is also associated with several shortcomings. For instance, studies using Pulse Field Gel Electrophoresis (PFGE) demonstrate that the initial DSB produced are quickly repaired with a half time of ∼20 min, with only a few remaining after 1 h.^32,33^ However, γH2AX is a marker of the metabolic activities initiated to repair the damage, rather than a direct marker of DSB. Although a few laboratories are able to observe γH2AX foci within minutes post exposure, their detection is difficult at these early times due to their small size, and they are more reliably scored 15–30 min post exposure. Many laboratories report an initial increase in foci number with time, reaching a maximum ∼30–60 min after exposure, with a reduction being observed at later times. Therefore, the initial yield of DSB may be underestimated due to many of the DSB being repaired during this period; additionally the subsequent rate of DSB repair reported at these later times is often slower than the initial rate of DSB repair.^33–35^ It has been proposed that γH2AX may, in some cases, continue to mark the site following the initial rejoining of the break and therefore may not always represent a physical break.^33,36^ γH2AX also detects replication-induced DSB in S-phase cells which will contribute to the persistence or delayed response of the signal. Even non-exposed S-phase cells typically have a higher number of background foci than other phases due to endogenously induced damage.^26,37^ Replication-induced DSB are also very different chemically to double-ended DSB and potentially much more likely to be repaired; it is therefore not clear whether these secondary foci always reflect the presence of DSB.^37^ γH2AX may also in some circumstances be generated by DNA transcriptional activity.^38–40^ It is important to note that the sensitivity, reliability, and absolute yields obtained with this technique can vary significantly between laboratories depending on many factors such as staining protocol, cell type, cell cycle, temperature, and critically on the imaging and scoring criteria used. This is illustrated by the large variation in results obtained for the 20 patients by Fiecheter *et al.,*^8^ with the median number of γH2AX per cell varied from 0 to 0.661 pre-exposure and 0 to 1.065 post-exposure; in contrast, the mean number of foci per cell obtained for 45 patients by Brand *et al.*^22^ is significantly smaller and much more consistent, varying from ∼0.09 to 0.17 and consistent with background levels of <0.2 foci per lymphocyte reported for ionizing radiation biodosimeter studies.^31,41^

Flow cytometry can also be used to analyse γH2AX with the advantage that thousands of cells can be analysed very quickly, through measuring the total fluorescence intensity rather than detecting individual foci. In general though the sensitivity of this approach is lower than that achieved by scoring individual foci, in part due to background foci intensity having a wider inter-individual variability.^42,43^ As a result, this technique does not maintain the level of sensitivity required to be used as a biodosimeter of ionizing radiation exposure beyond the first hours.^41^ The flow cytometry results of Fiecheter *et al.*^8^ and Lancelloti *et al.*^7^ both show significant variation between individuals, with some showing an increase while others a decrease in response. The delayed enhancement in γH2AX expression reported by Lancellotti *et al.*^7^ at day 2 and 1 month post exposure is inconsistent with DSB being produced during imaging as the majority of DSB are reported to be repaired within hours and the limited sensitivity of the flow cytometry assay at extended times following exposure. This could reflect an enhancement in the background level of DSB production (or other events leading to increased γH2AX expression) post exposure. However, the time course of this increase is still greater than the delayed peak of several hours often observed following exposure of exponentially growing cells to genotoxic agents such as UV and alkylating agents.^44^

When assessing the relevance of the studies investigating the induction of DNA damage such as DSB, it is important to remember that the production of such lesions does not necessarily lead to long-term consequences. In general, the vast majority of DNA damage produced will be transient because of repair processes. As a result, it is the longer term consequences of this damage that are more critical, the unrepaired fraction and the resultant genetic modifications. A more relevant end point to study would be CA production. These have been extensively used (dicentric aberrations in particular) in peripheral blood lymphocytes for assessment of ionizing radiation, both as a biological dosimeter of exposure^45,46^ and as a marker of relative risk to different qualities of radiation [The effectiveness of different qualities/types of radiation (e.g. x-rays, α-particles, and neutrons) at inducing a given biological end point for a given absorbed dose can vary significantly.] exposure.^47,48^ In addition, significant evidence exists for the role of CA in early stages of cancer, with data showing that the frequency of CA in peripheral lymphocytes may be a predictor of cancer incidence in human populations.^49–52^ MN may also be used as measure of CA induction and may result from acentric chromosome/chromatid fragments or whole chromosomes failing to be incorporated into the daughter nuclei. Evidence indicates a link between increased MN and elevated cancer risks.^53^ In addition to the dicentric chromosome assay, the MN assay has, to a lesser extent, been established as a technique for biodosimety for ionizing radiation. These have required careful optimization of the sample preparation, data analysis protocols, and harmonization of procedures between laboratories to ensure consistency. However, the sensitivity of these techniques limits their usefulness as a way of retrospectively determining dose in exposures of the order of 100 mGy or above (>2 DSB per cell). Therefore, investigating the induction of CA or MN in situations where there are fewer DSB per cell becomes increasingly challenging, requiring more cells to be scored and great care to ensure reliability of the data. Dicentric aberrations and MN produced by ionizing radiation are essentially stable and can be detected for several months following irradiation, with their loss being directly related to the rate of turnover of cells within the blood. It is therefore interesting that although Simi *et al.*^9^ reported an increase in MN in lymphocytes in blood collected immediately after *in vivo* exposure, no difference was observed compared with controls at later collection times (72, 94, and 120 h). Although lymphocytes within the blood are typically in G0 phase, the reported dominance of chromatid aberrations reported by Lee *et al*.^19^ suggests that rearrangements occur mainly during S-phase. To investigate CA/MN induction, the cells were stimulated to divide. It is therefore possible that the DNA lesions resulting in CA/MN were produced during DNA replication, potentially by replication-induced breaks/DSB resulting from stalled replication forks. So rather than MRI directly producing DNA lesions, it may perturb the homeostasis within the cells for a period of time resulting in an enhancement of endogenous damage.

## Endogenous DNA damage

**Figure 1:**
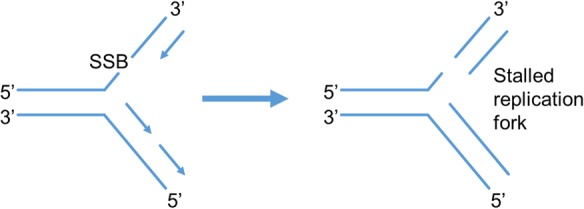
Schematic illustrating the production of stalled replication fork which can form γH2AX foci. A replication fork is formed during DNA replication, the two original stands branch forming single strands and serve as templates for synthesis of the complimentary strand which is synthesized in the 5′ to 3′ direction. The presence of a lesion, such as a SSB, can result in the replication fork stalling which can result in γH2AX foci.

DNA damage is a constant and a very frequent event in the daily life of all cells. Every cell within the body has a background level of the order of at least 50 000 endogenous DNA lesions per day as a result of reactive oxygen species (ROS) and other reactive metabolites, with the number expected to increase in situations with increasing oxidative stress.^54,55^ ROS include superoxide, hydrogen peroxide, hydroxyl radicals, and singlet oxygen and they can oxidize DNA, which can lead to several types of DNA damage chemically similar to those induced by ionizing radiation, although importantly their spatial and temporal distribution will be very different. These lesions are constantly being repaired with high fidelity by the cell via highly effective repair processes which help maintain genome integrity, minimizing the onset of ageing, and tumourigenesis. However, it has been proposed that these ubiquitous background events may account for an important fraction of oncogenic events in humans.^56^ Identification of these lesions and their production by free radical mechanisms have been the subject of numerous reviews.^57–60^ Although few, if any, DSB are formed by endogenous processes,^61,62^ events such as DNA damage lesions may lead to stalled replications forks during DNA replication (*Figure 1*) or interfere with DNA transcription, both of which can be detected by the γH2AX assay as mentioned earlier.^26,37,39,40^

It is well known that within the body cells do not act in isolation, with intercellular signalling being vital for maintaining the multicellular organization of tissue and the normal functioning of the constituent cells. ROS along with reactive nitrogen species (RNS) are formed as a consequence of both normal cell metabolism and inflammation and as a result of exposure to environmental factors. Free radicals and resulting reactive non-radical species are important for many physiological processes, including immunological defence, intra- and intercellular signalling, their concentration being determined by the balance between the rate of production and clearance by various antioxidants. As a result, there is a delicate balance between the advantageous and detrimental effects of these radicals.^63^ Environmental factors can lead to the activation of endogenous ROS or RNS-generating systems resulting in either a temporary or persistent dysregulation in signalling cascades and a change in ROS and/or RNS concentrations.^63^ This perturbation in homeostasis of this signalling and associated changes in oxidative stress can lead to a modulation of the background rate of endogenous DNA damage induction. For example, it is well known that very low doses of ionizing radiation can perturb intercellular signalling, levels of ROS, and other signalling molecules and can result in genotoxic effects such as induction of γH2AX foci, chromatid aberrations, MN, and apoptosis in unirradiated cells.^64–67^ Interestingly, rather than a continuous increase in effect with increasing exposure, the observed response is often consistent with a switch between two stable states essentially independent of triggering dose (if above a low threshold dose). It has been proposed that MR exposure may be able to increase the rate of production of DNA damage by endogenous processes as a result of an increase in oxidative stress.^68,69^

Assuming MRI can induce DNA lesions, the kinetics of induction and repair of any such lesions are also unclear from current studies but remain a crucial question that needs to be addressed. Are these lesions formed directly during imaging where some of the lesions may persist to later times, or are they formed through indirect processes that continue after imaging with a constant turnover as lesions are produced and repaired? This information would be helpful in guiding the understanding of the mechanisms, such as changes in homeostasis or persistent damage. A potential approach to address these questions would be to use live cell imaging using fluorescently labelled DNA-repair proteins and cell cycle markers, to identify the kinetics of formation and repair of individual lesions and cell cycle dependence. The relevance of any short-term increase in DNA damage as a result of an enhancement of endogenous processes has to be considered in the context that similar sites of DNA damage will be continuously produced throughout the lifetime of an individual and therefore any lesions, even if induced by MRI exposure, may not significantly add to the burden of risk associated with the background level of endogenous damage.

## Unique nature of DNA damage produced by ionizing radiation

The majority of recent experiments in investigating genotoxicity of MRI use the γH2AX assay as a measure of DSB. It is then tempting to compare induction of γH2AX and implied DSB yields produced following MRI with induction by imaging techniques using ionizing radiation.^3^ However, due to the way ionizing radiation interacts within the body, the resulting DNA damage produced includes damage unique to ionizing radiation, such as clustered DNA damage, and therefore, it is inappropriate to infer long-term health risks based simply on a comparison of yields of DSB or other non-DSB lesions. It is essential to deconvolute the γH2AX data into different classes of lesions and associated lifetimes, to be able to comment on the risks of MRI to humans.

**Figure 2:**
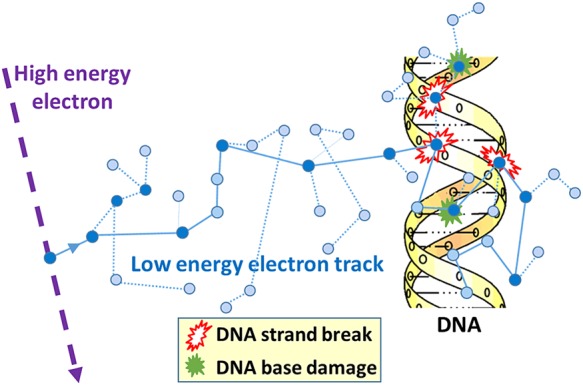
Schematic showing a typical track produced by the ubiquitous low-energy electron track-ends produced in a cell by ionizing radiation (dark circle = ionization event; light circle = excitation event). Individual DNA lesions are produced either by direct interaction with DNA or indirectly as a result of reactive radicals (most notably hydroxyl radicals) produced in the surrounding water (diffusion distance ∼6 nm). Clustered DNA damage, comprising combinations of two or more strand breaks and/or base damage, produced within one to two helical turns.

These assertions are founded on the knowledge that ionizing radiation interacts within the body and deposits energy along the resulting electron tracks producing highly structured tracks of ionization and excitation events that are stochastic in nature. This produces a wide variety of molecular damage, including base damage, abasic sites, SSB, and DNA–protein cross links. However, because these electrons produce multiple energy deposition sites along the resulting radiation track on the nanometre scale (correlated in time and space), it is capable of producing sites with multiple lesions on the DNA within a few base pairs of each other (*Figure 2*). It is the ability of ionizing radiation to produce clustered DNA damage which make it so biologically effective, with these longer lived clustered lesions dominating the long-term response rather than the much more numerous isolated DNA lesions that are chemically similar to those induced endogenously and are rapidly repaired. In mammalian cells, an exposure of 1 Gy of x-rays or γ-rays will produce ∼1300 base lesions, 1000 SSB, and 20–40 DSB (see *Table 2* for corresponding number for a typical chest CT scan). Although the number of lesions produced by ionizing radiation is significantly less than those produced daily by endogenous processes (∼50 000 per cell per day), it is the clustering of multiple lesions at a site of DNA damage^71,72^ and the corresponding reduced ability of their repair that is critical, leading to an increase in genetic modification (clustered lesions produced by endogenous damage are rare and do not match the degree of complexity that can be produced by ionizing radiation^73,74^). Although the majority of ionizing radiation-induced DSB will be repaired within ∼60 min, a small fraction may persist for >24 h.^75–78^ Theoretical analysis of these DSB using Monte Carlo calculations^79–82^ indicates that 20–50% of these DSB are in fact complex DSB (with extra single-strand breaks and/or associated base damage within a distance of 10 base pairs).^80^ In fact, the frequency and complexity of DSB typically increase with ionization density of the radiation and are important factors in accounting for differences in relative biological effectiveness (RBE) with radiation quality.^80^ This is also reflected in the variation in biological efficiency per DSB produced as a function of radiation quality, with even the DSB produced by sparsely ionizing x-rays estimate to be 4–40 times more effective than simple DSB produced by high concentrations of hydrogen peroxide.^83^ Additionally, as DSB are formed along the path of the resulting electron tracks following exposure, the DSB produced can be correlated in space and time increasing the chance that they may interact and lead to genetic and chromosomal rearrangements.^84^

**Table 2:** Yield of major lesions induced per mammalian cell following a 1 Gy exposure of x-rays (adapted from Cadet *et al.*^58^ and Lomax *et al.*^70^) and corresponding number for exposed cell following a typical adult abdominal CT

Radiation-induced DNA lesions	Number/Gy/cell	Adult abdominal CT (10 mGy to stomach)
Pyrimidine lesions	850	8.5
Purine lesions	450	4.5
Single-strand breaks (SSB)	1000	10
Double-strand breaks (DSB)	20–40	0.2–0.4

In addition to DSB and complex DSB, ionizing radiation will also form non-DSB clustered lesions. These non-DSB clustered lesions formed contain two or more lesions within the order of 10 base pairs and may occur either on the same strand (tandem) or opposite strands (bistranded). Complex non-DSB damages have been shown to make up a significant component of the lesions induced by radiation, being at least 4–8 times greater in number than prompt DSB for x-rays and γ-rays.^71,72,85–88^ While isolated lesions (e.g. base damage or SSB) are repaired quickly with high fidelity, for non-DSB clusters the rate of repair is typically impaired by the presence of additional lesions within the cluster.^89–91^ The extent of the reduction is dependent on the types and number of lesions along with their separation and orientation.^89^ Although processing of certain bistranded lesions may in a few cases lead to repair-induced DSB, the most likely consequence of these non-DSB clustered lesions will be an increase in their lifetime, and therefore, an increased chance of resulting in a stalled replication fork and potentially lead to replication-induced DSB in S-phase (*Figure 1*). It has been estimated that ∼10% of non-DSB clustered lesions will be converted to DSB as a result of processing,^32^ with the possibility that some of these DSB may be complex by virtue of additional lesions close by.^72,92^ Experiments have shown that these clustered lesions result in an increase in mutation frequencies in bacteria, yeast, and mammalian cells.^89,93,94^

Little is known about the mechanisms by which MRI can produce DNA damage, if indeed it does. It is know that the fields associated with MRI are non-ionizing and therefore unable to produce free electrons with sufficient energy to produce clustered damage sites over 1–2 turns of the DNA, similar to that associated with ionizing radiation. Therefore, any lesions that are produced as a result of MRI are much more likely to be repaired with high fidelity than those produced by ionizing radiation.

## Conclusions

Although MRI imaging is generally considered to be safe compared with imaging technology using ionizing radiation, there is increased concern about the potential long-term health effects of exposure, especially with the push to higher static fields along with stronger and faster switching fields. Although IARC^15^ did not find clear evidence that SMF are carcinogenic, the data are limited, underpowered, or suffer methodological weakness.^11^ Both IARC and the recent SCENIHR^14^ report suggest the need for a large, carefully designed epidemiology study.

The ability of MRI to produce DNA damage and potential mechanisms involved remains unclear. Generally no consistent response has been reported between the very limited number of published studies, with limited or no controls, typically based on data from a small number of subjects or often a single experiment and therefore limited statistical power. It is therefore essential that future studies employ strict standardization in experimental design and scoring criteria, along with quality control measures to ensure reproducibility and consistency. All techniques should be carefully benchmarked against negative and positive controls (potentially using ionizing radiation) to confirm the validity and reproducibility of the results, collected over multiple independent experimental measurements and ideally include inter-laboratory comparisons. There must also be a willingness by the scientists and journals to publish negative results for well-designed experiments. While there is a tendency to focus on the initial induction of DNA damage, it is more relevant to study downstream effects, for example CA production for which there is some evidence to this could be a marker of cancer risk. If a reproducible effect can be observed, then these studies will also need to critically assess the importance of contrast agents, along with potential confounding factors and other potential synergistic effects (such as smoking, medication, health issues relating to reason for the scan, and underlying genetic sensitivity). An additional issue to be addressed would be to determine the critical component of exposure (SMF, GMF, RF) if possible to compare responses for different scans between laboratories.

It is important that great care is taken to ensure that any potential genetic effects associated with MRI exposure, if it exists, are not over interpreted. There is a danger that in the absence of clear evidence, physicians and patients are misinformed by the presentation of preliminary data, affecting their choice of potentially life-saving imaging tests. The ability for MRI to produce DNA lesions has yet to be robustly demonstrated. If lesions are formed, then the relative probability of these lesions to produce long-term health effects has to be put in the context of the risk associated with the 50 000 lesions produced daily by endogenous processes. As discussed, the fields associated with MRI are non-ionizing, having insufficient energy to directly break chemical bonds and are thus unable to produce free electrons with sufficient energy to produce clustered lesions including complex DSB with an associated reduction in repairability. The complex DSB produced by ionizing radiation have been shown to be more effective than simple DSB at producing a wide range of biological end points, including mutations, with a greater probability of leading to long-term health effects. It is therefore important not to try an infer cancer risk by simply comparing the initial yields of DSB produced by MRI to that produced by a given exposure of ionizing radiation. The γH2AX assay not only marks classical double-ended DSB, but also stalled replication forks and/or collapse in S-phase or interfere with DNA transcription.^37,40^ It would also be appropriate to investigate how imaging may perturb intercellular signalling and associated levels of oxidative stress which may lead to modulation in endogenous damage levels, epigenetic changes, and genomic stability in cells and their progeny. In summary, what the field needs at the moment is more research and less fear mongering.
